# Crosstalk between Dpp and Tor signaling coordinates autophagy-dependent midgut degradation

**DOI:** 10.1038/s41419-019-1368-9

**Published:** 2019-02-08

**Authors:** Donna Denton, Tianqi Xu, Sonia Dayan, Shannon Nicolson, Sharad Kumar

**Affiliations:** Centre for Cancer Biology, University of South Australia and SA Pathology, GPO Box 2471, Adelaide, SA 5001 Australia

## Abstract

The majority of developmentally programmed cell death (PCD) is mediated by caspase-dependent apoptosis; however, additional modalities, including autophagy-dependent cell death, have important spatiotemporally restricted functions. Autophagy involves the engulfment of cytoplasmic components in a double membrane vesicle for delivery to the lysosome. An established model for autophagy-dependent PCD is *Drosophila* larval midgut removal during metamorphosis. Our previous work demonstrated that growth arrest is required to initiate autophagy-dependent midgut degradation and Target of rapamycin (Tor) limits autophagy induction. In further studies, we uncovered a role for Decapentaplegic (Dpp) in coordinating midgut degradation. Here, we provide new data to show that Dpp interacts with Tor during midgut degradation. Inhibiting Tor rescued the block in midgut degradation due to Dpp signaling. We propose that Dpp is upstream of Tor and down-regulation promotes growth arrest and autophagy-dependent midgut degradation. These findings underscore a relationship between Dpp and Tor signaling in the regulation of cell growth and tissue removal.

## Introduction

Programmed cell death (PCD) is essential for animal life and in most contexts is mediated by caspase-dependent apoptosis^1^. Multiple additional modes of PCD have recently come to light^2^. One such modality is autophagy-dependent cell death, which plays important spatiotemporal restricted functions^3–5^. Several context-specific examples of autophagy in cell death have been identified in *Drosophila* including the removal of obsolete larval tissues, including the midgut, during metamorphosis^3,5,6^. The larval midgut is a large tissue with anterior appendages called gastric caeca and in response to a pulse of the steroid hormone ecdysone at the larval–pupal transition, midgut PCD initiates with the initial contraction of the gastric caeca and the condensation of the gut^7,8^. Degradation of the larval midgut does not require caspase-dependent apoptosis but occurs by an autophagy-dependent cell death mechanism^6^.

Autophagy is important for normal development, cellular homeostasis, metabolism, cell growth, and cell death^9^. Basal levels of autophagy are required under growth conditions to maintain cellular homeostasis, and in response to various stress and extracellular cues high levels of autophagy are induced. The induction of autophagy occurs in response to upstream signaling pathways that converge on Target of rapamycin (TOR) kinase, as part of a multi-protein complex TORC1^10^. In the presence of nutrients and growth signals TORC1 activity negatively regulate autophagy phosphorylating and inhibiting Atg1/Unc51-like kinase 1 (Ulk1) complex activity^11^. Under growth-limiting conditions such as starvation, TORC1 is no longer active enabling autophagy induction by Atg1 activation promoting the initiation of autophagosome formation^12^.

Degradation of the *Drosophila* larval midgut is triggered by an increase in the steroid hormone ecdysone. In addition to the hormonal cue, down-regulation of growth signaling and TORC1 activity precedes autophagy-dependent midgut degradation^13,14^. Similar to conditions of nutrient limitation where TORC1 inactivation promotes autophagy induction, ablation of *Tor* and *raptor* (but not TORC2 component rictor) promotes premature autophagy-dependent midgut degradation^14,15^. While TORC1 is critical for autophagy regulation, the interplay with the signals upstream of Tor in the regulation of autophagy-dependent cell death remains poorly understood. In recent studies we identified Decapentaplegic (Dpp), the *Drosophila* bone morphogenetic protein/transforming growth factor β ligand, in the regulation of autophagy-dependent midgut degradation^16^. To understand the crosstalk between these pathways in regulating autophagy-dependent midgut PCD, in this report we have investigated epistasis between the Dpp pathway and Tor.

## Results and discussion

### Dpp expression prevents autophagy and midgut degradation

**Fig. 1:**
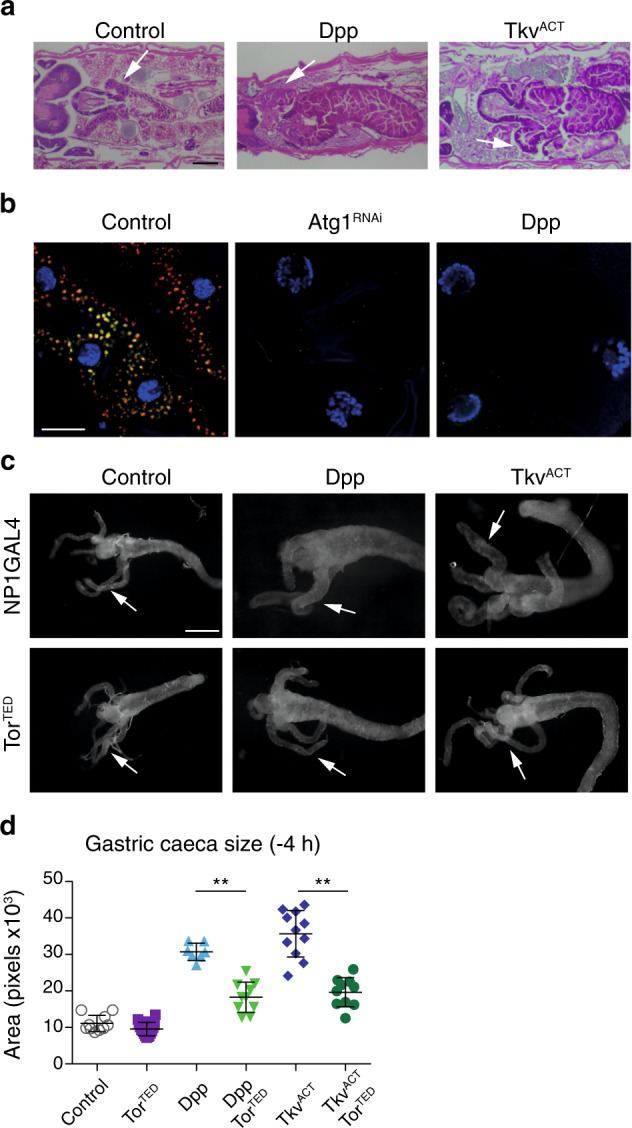
Dpp blocks autophagy and midgut degradation. **a** Expression of Dpp and Tkv^ACT^ prevents midgut removal. Histology from control *(NP1-GAL4/+)*, Dpp *(NP1-GAL4/+; UAS-dpp/+)* and Tkv^ACT^ *(NP1-GAL4/+; UAS-tkv*^*ACT*^*/+)* midguts from late third instar animals (−4 h RPF) shows enlarged midgut and gastric caeca (arrows). Scale bar represents 200 μm. **b** Autophagy flux visualized using GFP-mCherry-Atg8a in midgut cells from control *(NP1-GAL4/UAS-GFP-mCherry-Atg8a)*, *Atg1*^*RNAi*^ *(NP1-GAL4/UAS-GFP-mCherry-Atg8a; UAS-Atg1*^*RNAi*^*/+)*, and Dpp *(NP1-GAL4/UAS-GFP-mCherry-Atg8a; UAS-dpp/+)* larvae at −4 h RPF. Scale bar represents 20 μm. **c** Expression of dominant-negative TOR (Tor^TED^) suppresses the phenotype of of Dpp and Tkv^ACT^. Morphology from *Tor*^TED^ (*NP1-GAL4/UAS-Tor*^*TED*^) with the co-expression of Dpp *(NP1-GAL4/UAS-Tor*^*TED*^*; UAS-dpp/+)* and Tkv^ACT^ *(NP1-GAL4/UAS-Tor*^*TED*^*; UAS-tkv*^*ACT*^*/+)* midguts from −4 h RPF shows contracted gastric caeca (arrows). Scale bar represents 200 μm. **d** Quantification of gastric caeca size (data represent as the average pixels ± SD; **p < 0.0001)

A complex interplay between hormonal cues and growth signaling pathways is important for the initiation of autophagy-dependent cell death. To dissect out the regulatory mechanisms we identified *dpp* as a novel regulator of autophagy-dependent PCD^16^. Expression of Dpp in the midgut using the NP1-GAL4 driver resulted in enlarged midguts that do not contract like the control midguts (Fig. 1a). These animals fail to undergo metamorphosis and die as late third instar larvae^16^. The Thickveins (Tkv) receptor is required for Dpp signaling and ligand independent signaling can be achieved by expression of a constitutively active receptor Tkv^Q253D^ (Tkv^ACT^)^17^. Similar to Dpp, expression of Tkv^ACT^ using NP1-GAL4 resulted in enlarged midguts and larval lethality (Fig. 1a).

Induction of autophagy results in association of Atg8a with autophagosomal membranes that can be observed as puncta. To examine autophagy flux in whole midguts we examined GFP-mCherry-Atg8a puncta formation. This revealed that similar to *Atg1* knockdown, Dpp expression completely blocked induction of autophagy (Fig. 1b)^16^. While the control midguts showed strong induction of autophagy as indicated by the presence of both red and yellow puncta, thus quenching of the GFP signal in the autolysosome, both *Atg1* knockdown and Dpp overexpression lacked any puncta (Fig. 1b). These results confirm that sustained Dpp signaling prevents autophagy and midgut size contraction, suppressing developmental PCD^16^.

### Dpp signaling interacts with Tor signaling

Autophagy is maintained at basal level under growth conditions through upstream signaling pathways that converge on TOR kinase^12^. A key first step in autophagy induction is activation of a multi-protein complex containing Atg1, which is inhibited by active TOR. In previous studies we have shown that depletion of *Atg1* and *Atg18* in the midgut blocks autophagy and severely delays PCD^6^. Conversely, knockdown of *Tor* results in premature autophagy induction and midgut PCD^14^. To understand the mechanism(s) by which Dpp signaling regulates midgut removal we examined if there is an interaction between Tor and Dpp.

**Fig. 2:**
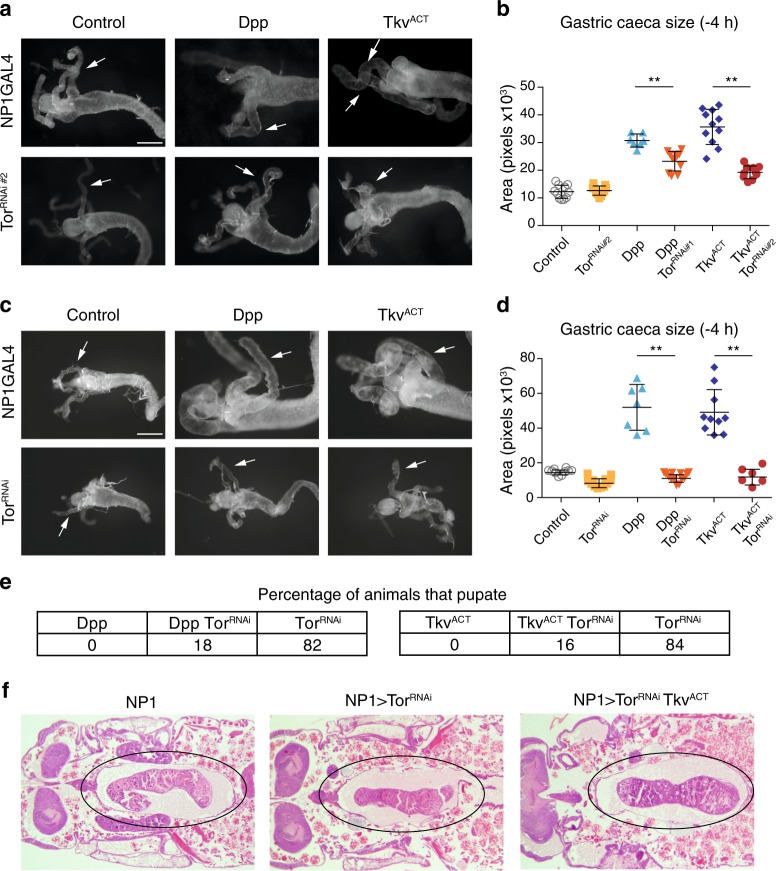
Genetic interactions between Dpp signaling and Tor. Knockdown of *Tor* suppresses the phenotype of Dpp and Tkv^ACT^. **a** Morphology from *Tor*^*RNAi*^ knockdown line #2 (*NP1-GAL4/+; UAS-Tor* ^*RNAi#2*^*/+*) with the expression of Dpp *(NP1-GAL4/+; UAS-dpp/UAS-Tor*^*RNAi#2*^*)* and Tkv^ACT^ *(NP1-GAL4/+; UAS-Tor*^*RNAi#2*^*/UAS-Tkv*^*ACT*^*)* midguts from −4 h RPF shows contracted gastric caeca (arrows). Scale bar represents 200 μm. **b** Quantification of gastric caeca size (data represent as the average pixels ± SD; **p < 0.0001). **c** Morphology from a second independent *Tor*^*RNAi*^ knockdown line (*NP1-GAL4/+; UAS-Tor* ^*RNAi*^*/+*) with the expression of Dpp *(NP1-GAL4/+; UAS-dpp/UAS-Tor*^*RNAi*^*)* and Tkv^ACT^ *(NP1-GAL4/+; UAS-Tor*^*RNAi*^*/UAS-Tkv*^*ACT*^*)* midguts from −4 h RPF shows contracted gastric caeca (arrows). Scale bar represents 200 μm. **d** Quantification of gastric caeca size (data represent as the average pixels ± SD; **p < 0.0001). **e** Histology sections from +12 h RPF show the knockdown of *Tor*^*RNAi*^ with Tkv^ACT^ expression promotes animal survival with midgut contraction similar to controls (circled). **f** Number of animals that pupated from combined *Tor*^*RNAi*^ knockdown with either expression of Dpp or Tkv^ACT^

Initially, we examined the consequence of simultaneous expression of a dominant-negative Tor (Tor^TED^) with Dpp and Tkv^ACT^ expression. Interestingly expression of Tor^TED^ was sufficient to significantly suppress both the Dpp and Tkv^ACT^ midgut phenotypes (Fig. 1c, d). The block in gastric caeca contraction due to Dpp or Tkv^ACT^ expression was rescued by expression of Tor^TED^ to a size similar to the control. We then examined the consequence of simultaneous ablation of *Tor* with Dpp and Tkv^ACT^ expression. We have previously shown the level of knockdown for two independent *Tor* RNAi lines, with one line (*Tor*^*RNAi*^) providing greater knockdown than a second line (*Tor*^*RNAi#2*^*)*^14^. Consistent with Tor^TED^ expression, the knockdown of *Tor (Tor*^*RNAi#2*^*)* significantly suppressed both the Dpp and Tkv^ACT^ midgut phenotypes (Fig. 2a, b). This finding was further supported by the use of the independent RNAi knockdown line that provides stronger knockdown, which also showed that *Tor* knockdown is a suppressor of the Dpp and Tkv^ACT^ midgut phenotypes (Fig. 2c, d). The expression of Dpp and Tkv^ACT^ in the midgut causes a developmental arrest prior to the onset of metamorphosis; however, the knockdown of *Tor* rescued the block in midgut degradation by Dpp and Tkv^ACT^ and promoted animal survival to a later stage of development (Fig. 2e). Strikingly, the Tkv^ACT^ animals survived until +12 h Relative to puparium formation (RPF) and examination of the midgut at this later stage revealed the tissue had undergone contraction similar to the control (Fig. 2f). This suggests that the suppression of Tkv^ACT^ by *Tor* knockdown was maintained. These data indicate an interaction between Tor and Dpp signaling pathways and are consistent with the down-regulation of Dpp signaling required for autophagy-dependent midgut removal^16^.

**Fig. 3:**
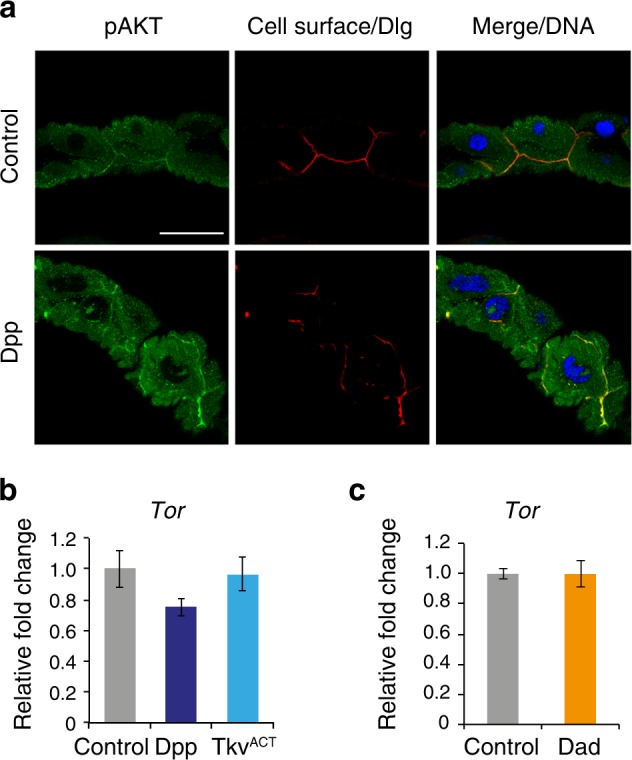
Dpp activity does not alter growth signaling or *Tor* levels. **a** Cortical localization of phospho-Akt (green, arrow) adjacent to Dlg (red), a cell surface marker, in control (*NP1-GAL4/+*) and Dpp (*NP1-GAL4/+; UAS-Dpp/+*) midgut at −4 h RPF. **b** The level of *Tor* transcripts from larval midgut expressing Dpp or Tkv^ACT^ was similar to control. Transcript level of *Tor* was measured by qRT-PCR from control, *NP1* *>* *dpp* and *NP1* *>* *tkv*^*ACT*^ larval midguts at −4 h RPF. **c** The level of *Tor* transcripts from larval midgut expressing Dad was similar to control. Transcript levels were measured by qRT-PCR from control and *NP1* *>* *Dad* larval midguts at −4 h RPF. **b**, **c** Data are from three experiments, with 20 midguts per sample (average ± SEM)

Given that the midgut phenotype due to Dpp expression could be suppressed by ablation of *Tor*, we examined if growth signaling and *Tor* levels were altered in response to Dpp signaling. Under growth conditions PI3K activates Tor thus inhibiting autophagy and down-regulation of PI3K leads to Tor inactivation promoting autophagy^12^. Growth signaling can be monitored by PI3K activity through the localization of phosphorylated Akt to the cell cortex, owing to its interaction with PIP3, and its subsequent phosphorylation that is required for downstream signal transduction^13^. To further investigate if Dpp expression perturbs growth signaling in the midgut we examined the localization of phosphorylated Akt, compared with the cell cortex marker Dlg. In both control and Dpp-expressing midguts during late larval stages (−4 h RPF), phosphorylated Akt was detected at the cell cortex (Fig. 3a). Furthermore, there was no significant change in the *Tor* transcript levels either in the presence (Dpp and Tkv^ACT^ expression) (Fig. 3b) or absence (expression of inhibitory Smad, Dad) of Dpp activity (Fig. 3c). Together the data suggest that in the presence of Dpp activity growth signaling is similar to the control and is not promoting increased *Tor* expression.

### Tor knockdown restore autophagy in Dpp expressing midguts

**Fig. 4:**
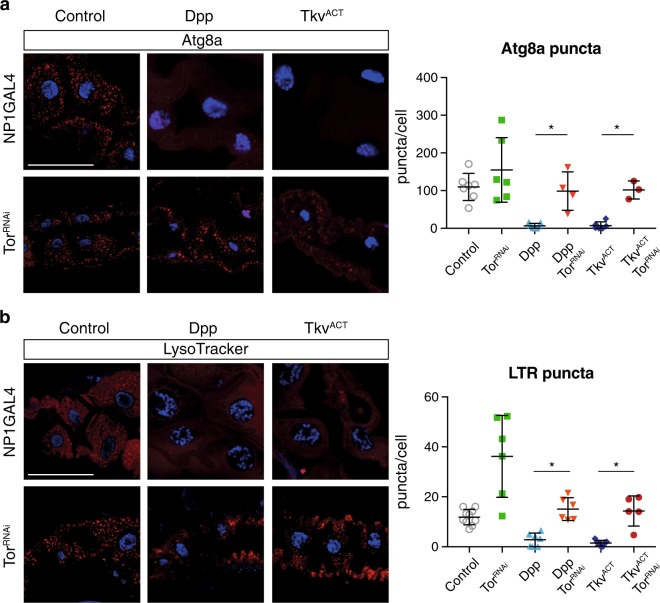
Knockdown of *Tor* restores autophagy in the presence of Dpp. **a** Autophagy detected by Atg8a puncta in midgut cells from Dpp *(NP1-GAL4/+; UAS-dpp/+)* and Tkv^ACT^ *(NP1-GAL4/+; UAS-tkv*^*ACT*^*/+)* is greatly reduced compared to the control *(NP1-GAL4/+)* at −4 h RPF. Increased Atg8a puncta is observed in *Tor*^*RNAi*^ knockdown alone and when combined with Dpp *(NP1-GAL4/+; UAS-dpp/UAS-Tor*^*RNAi*^*)* and Tkv^ACT^ *(NP1-GAL4/+; UAS-Tor*^*RNAi*^*/UAS-Tkv*^*ACT*^*)*. Quantification of Atg8a puncta measured using ImageJ (data represent as the average puncta/cell ± SD; **p < 0.05). **b** LysoTracker Red shows low basal levels in Dpp and Tkv^ACT^ midguts at −4 h RPF. Increased LysoTracker Red staining is observed in *Tor* knockdown alone and when combined with Dpp *(NP1-GAL4/+; UAS-dpp/UAS-Tor*^*RNAi*^*)* and Tkv^ACT^ *(NP1-GAL4/+; UAS-Tor*^*RNAi*^*/UAS-Tkv*^*ACT*^*)*. Quantification of LTR puncta measured using ImageJ (data represent as the average puncta/cell ± SD; *p < 0.05). Scale bar represents 100 μm (**a**, **b**)

Dpp expression prevents induction of autophagy and *Tor* ablation induces premature autophagy. To determine if the phenotypic rescue was due to induction of autophagy, we examined autophagy using Atg8a and LysoTracker staining. While the Dpp and Tkv^ACT^ midguts show very little Atg8a staining, when combined with reduced *Tor* levels Atg8a puncta can be observed (Fig. 4a). Although not a direct marker of autophagy, LysoTracker staining has also been used to detect autophagy-associated lysosomal activity in the fat body and midgut^14,15^. Such staining showed that reduction of *Tor* levels in the Dpp and Tkv^ACT^ midguts was sufficient to restore LysoTracker-positive vesicles indicating increased autophagy flux (Fig. 4b). Together, these findings indicate that reducing *Tor* levels is sufficient to restore autophagy flux in the Dpp and Tkv^ACT^ expressing midguts.

**Fig. 5:**
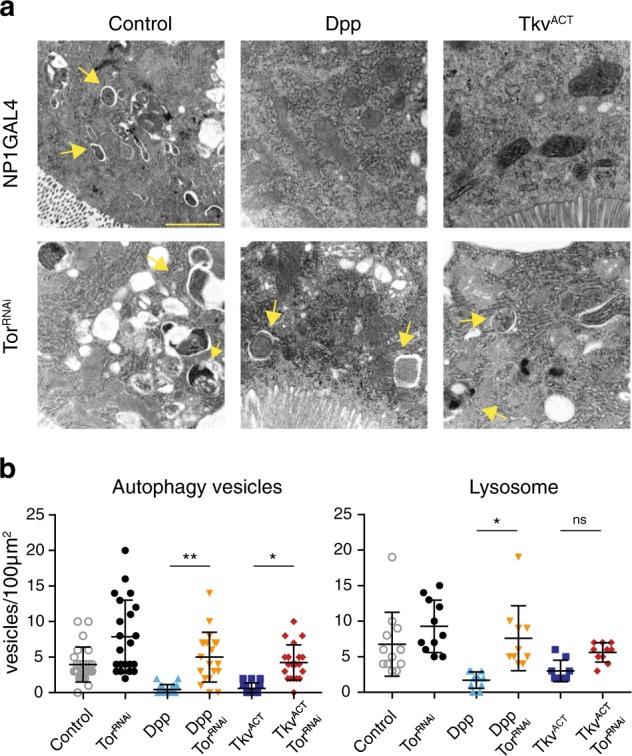
Autophagic vesicles are present in Dpp when Tor is knocked-down. **a** Representative TEM images from sections of midgut at −4 h PRF. Control *(NP1-GAL4/+)* and *Tor*^*RNAi*^ cells possess autolysosomal structures (arrows); Dpp and Tkv^ACT^ midgut cells lack structures. Autolysosomal structures can be detected in the double *Tor*^*RNAi*^ knockdown with Dpp or Tkv^ACT^ (arrows). **b** Quantitation of the number of autophagic vesicles (autophagosomes and autolysosomes) and lysosomes (data represented as average vesicle/area ± SD; **p *<* 0.0001, *p < 0.01, ns not significant). Scale bar represent 1 μm

To further investigate the effects of Dpp and *Tor* on autophagy we used transmission electron microscopy (TEM). The data showed that Dpp and Tkv^ACT^ midgut cells have very few autophagosomes or autolysosomal structures (Fig. 5a, b). A large number of autophagosomes and autolysosomes could be identified when *Tor* was knockdown (Fig. 5a, b). This analysis also revealed that midgut cells with combined *Tor* knockdown and Dpp or Tkv^ACT^ expression contained more autophagic vesicles compared to cells expressing Dpp or Tkv^ACT^ alone (Fig. 5a, b). Quantitation of the number of autophagic vesicles and lysosomes is consistent with the rescue of midgut degradation and increase in autophagy markers (Fig. 5b). Together, these data indicate an interaction between Tor and Dpp pathways, whereby down-regulation of Dpp signaling is required for autophagy-dependent midgut removal.

In addition to hormonal cues, the down-regulation of growth signaling is important for the induction of autophagy-dependent cell death in the midgut^13^. During larval development, down-regulation of Tor signaling in the midgut promotes autophagy and midgut removal^14^. Similarly, down-regulation of PI3K and Ras signaling are required for proper midgut removal^13^. In addition, Dpp plays an important role in autophagy-dependent midgut degradation, whereas other morphogens including Hh and Wg are not required.^18^ Our data presented here indicate that induction of autophagy by *Tor* depletion rescues the effect of Dpp signaling. This supports our recent findings that Dpp signaling prevents midgut removal by blocking autophagy induction^16^. Together these findings establish new connections between Dpp and Tor signaling pathways during autophagy-dependent midgut cell death. It will be important to understand how Dpp and Tor signals are integrated with other growth signals in the midgut and what triggers their down-regulation to promote midgut degradation.

## Materials and methods

### Fly stocks

The midgut driver *P{GawB}Myo31DFNP0001* referred to as *NP1-GAL4* was obtained from the Drosophila Genetic Resource Center (Kyoto, Japan). The following stocks were from the Bloomington Drosophila Stock Center (Bloomington, IN, USA) including RNAi lines from the Transgenic RNAi Project (http://www.flyrnai.org): *w*^*1118*^, *Atg1*^*RNAi*^ *(y*^*1*^ *v*^*1*^*; P{TRiP*.*JF02273}attP2)*, *Tor*^*RNAi*^ *(y*^*1*^ sc* v^*1*^*; P{TRiP*.*HMS00904}attP2;* strong*)*, *Tor*^*RNAi*^*#2 (y*^*1*^ sc* v^*1*^*; P{TRiP*.*GL00156}attP2;* weak*)*, *UAS-Tor*.*TED*, UAS-dpp (w*; UAS-dpp.*S 42B)*, *UAS-tkv*^*ACT*^ (w*; UAS-tkv.*Q253D/TM3*, *Sb1 Ser1)*, *and UASp-GFP-mCherry-Atg8a*. The control was *w*^*1118*^ crossed to *NP1-GAL4*. All flies were maintained and crosses performed at 25 °C on cornmeal, molasses, and yeast medium.

### Larval staging and midgut morphology analysis

To stage larvae 0.05% bromophenol blue was added to food and wandering third instar larvae were transferred to a Petri dish lined with moist Whatmann paper to monitor for clearance of blue food in the gut^19^. The morphology of the midgut was examined from a minimum of 10 appropriately staged animals by dissection in phosphate-buffered saline (PBS), then fixed in 4% formaldehyde/PBS, and imaged using a stereozoom microscope (Olympus, Tokyo, Japan). The size of the gastric caeca was measured from these images in Photoshop (Adobe, San Jose, CA, USA) using the magnetic lasso tool and histogram function to determine pixels in the area as described previously^13^.

### Histology

For whole animal sections 10–15 animals from −4 h RPF larvae or +12 h RPF pupae were fixed in FAAG (85% ethanol, 4% formaldehyde, 5% acetic acid, and 1% glutaraldehyde), then paraffin embedded prior to sectioning and hematoxylin and eosin staining as previously described^6^.

### Live imaging

To image fluorescently tagged GPF-mCherry-Atg8a midguts were dissected in PBS with Hoechst 33342 (Sigma-Aldrich), mounted in PBS, and imaged immediately without fixation using a Zeiss LSM 700 or 800 confocal microscope (Detmold Imaging Core Facility, SA Pathology, Adelaide, SA, Australia). For LysoTracker staining, midguts were dissected in PBS and transferred to staining solution containing 100 nM LysoTracker Red DND-99 and 1 μg/ml Hoechst 33342 in PBS and then incubate in the dark for 2–5 min at room temperature. Samples were then washed with PBS for 5 min, mounted in PBS, and imaged immediately without fixation. Quantitation of images was achieved using ImageJ to count the number of puncta per cell (with a size larger than 2 pixels).

### Immunohistochemistry

Midguts were dissected in PBS, fixed in 4% paraformaldehyde in PBS for 20 min at room temperature, and blocked with 5% normal goat serum as described. Primary antibodies used were rabbit anti-GABARAP1 (referred to as Atg8a) (1:200) (Abcam, Cambridge, MA, USA), rabbit anti-phospho-Drosophila-Akt (1:200) (Cell Signaling, Danvers, MA, USA), and mouse anti-Dlg (1:100) (4F3 anti-discs large was deposited by Goodman, C.; Developmental Studies Hybridoma Bank, Iowa, IA, USA). Secondary antibodies used were anti-rabbit Alexa-FLUOR 488 (Molecular Probes, Eugene, CA, USA), anti-rabbit Alexa-FLUOR 568 (Molecular Probes), and anti-mouse Alexa-FLUOR 568 (Molecular Probes). Hoechst 33342 (Sigma-Aldrich, St. Louis, MO, USA) was used to detect DNA. The samples were mounted in 80% glycerol in PBS and imaged using a Zeiss LSM 700 confocal microscope.

### Confocal imaging

Confocal images were obtained at room temperature using a Carl Zeiss LSM 700 or Zeiss LSM 800 inverted confocal microscope (Zeiss Laboratories) with 405 nm (5 mW), 488 nm (10 mW), and 555 (10 mW) lasers and C Apo ×40/1.2 W DICII objective. The dual labeled samples were imaged with two separate channels (PMT tubes) in a sequential setting. On the LSM 700, Zen gray was used to capture the images and on LSM 800 images were captured and Airyscan processed using Zen blue. All images were then processed using Photoshop (Adobe).

### Transmission electron microscopy

Midguts were dissected in PBS, fixed in 1.25% glutaraldehyde, 4% sucrose, 4% paraformaldehyde in PBS for 30 min at room temperature and then washed with 4% sucrose in PBS. Samples were then post-fixed in 1% osmium tetroxide for 1 h, dehydrated in ethanol, treated with propylene oxide for 15 min, and infiltrated for embedding in resin as described.^20^ Ultrathin sections were cut on grids, stained with 4% uranyl acetate in 25% ethanol and Reynold’s lead citrate before imaging using Tecnai G2 Spirit TEM (Adelaide Microscopy).

### Quantitative real-time PCR (qRT-PCR)

Total RNA was isolated from 20 midguts/sample using TRIzol reagent (Invitrogen). cDNA synthesis was performed using High Capacity cDNA Reverse Transcription Kit (Applied Biosciences, Life Technologies, Carlsbad, CA, USA) with random primers and 1 μg of total RNA. qRT-PCR was performed on a Rotor-Gene Q (Qiagen, Valencia, CA, USA) with Rotor-Gene software (version 2.1.0.9) using KAPA SYBR® FAST according to the manufacturer’s instructions. Reactions were performed using three independent biological samples in triplicate and transcript levels were normalized using rp49 as the reference gene. Data were analyzed using the Q-Gene software with Standard Curves, and samples on the same graph were run simultaneously as described in ref. ^16^. Primers used are as follows:

*Tor* F 5′-CGGTTATCCCGCTCAGTACC; R 5′-GGTGATCATAGTCTGGCGCA

*rp49* F 5′-CCAGTCGGATCGATATGCTAA; R 5′-ACGTTGTGCACCAGGAACTT

### Statistical analysis of data

The statistical analysis performed on the quantitation data was an ordinary one-way analysis of variance with Tukey’s multiple comparisons test using Prism (GraphPad Software) and data are expressed as mean ± SD. Any images where the cell number could not be accurately determined were excluded from quantitation.
